# Home‐Based Parenting Strategies and Challenges of Primary Parental Caregivers of Children With Medical Complexity in Japan: A Qualitative Study

**DOI:** 10.1111/cch.70308

**Published:** 2026-06-28

**Authors:** Miku Yamaguchi, Yuki Suzuki

**Affiliations:** ^1^ Department of Pediatric Nursing Kyoto Prefectural University of Medicine School of Nursing Kyoto Japan; ^2^ Department of Child Health Nursing, Faculty of Health Care and Nursing Juntendo University Urayasu Japan

**Keywords:** care coordination, children with medical complexity, health services use, parental caregivers, qualitative research, respite

## Abstract

**Background:**

In Japan, the number of children with medical complexity (CMC) is increasing. In response, the Act on Support for Children Requiring Constant Medical Care and Their Families mandated prefectural support centre establishment and promoted coordination across health, welfare and education sectors. In addition to services comparable to those in the United States (e.g., public school options and respite care), Japan strengthened publicly funded home‐visit physician, nursing, and personal helper and after‐school services to support parents. However, it remains unclear how parents adapt to this evolving system. This study aimed to qualitatively examine the strategies and challenges of primary parental caregivers of CMC in Japan to generate insights that can support family‐centred services and clinical care practice.

**Methods:**

We conducted a qualitative study guided by the Consolidated Criteria for Reporting Qualitative Research and included 13 primary parental caregivers. The inclusion criterion was ≥ 1 year as the principal at‐home caregiver of CMC (< 18 years).

**Results:**

Interviews were transcribed verbatim and inductively coded by two experienced researchers to achieve thematic saturation. Caregivers were predominantly mothers (11 mothers [female] and 2 fathers [male]). The median [interquartile range] ages of the parents and children were 47.0 [35.0–50.0] and 10.0 [7.0–13.0] years, respectively. Eight themes were identified. The challenge subthemes highlighted insufficient paediatric‐trained home‐visit nursing services, weak support for interagency coordination and transition‐to‐adult care support and inadequate respite care as priority areas for healthcare professionals.

**Conclusions:**

Parents effectively leveraged supportive policies; however, deficiencies remain in paediatric‐trained home‐visit nursing services, interagency coordination, transition‐to‐adult care support and respite care. These challenges mirror international reports indicating global and implementation‐level gaps. Additionally, reflecting on the unique context of Japan, caregiving was predominantly mother‐centred, and practical strategies for disaster preparedness were identified. Family‐centred care should prioritize workforce expansion, stronger cross‐sector coordination and practical respite solutions at the point of care.

## Summary

- Children with medical complexity (CMC) constitute a distinct paediatric group marked by chronic multisystem conditions, technology dependence, high healthcare use and heavy caregiving needs.
- The Japanese government has strengthened publicly funded home‐visit physician and nursing services, home‐visit personal helpers and after‐school day services to support CMC and their parents.
- Despite Japan's strengthened policy framework, the challenges identified were largely shared with those in other countries.
- Expand the paediatric‐trained home‐visit nursing workforce; strengthen transition‐to‐adult care is a clinical priority.
- Enhance coordination among home‐ and community‐based agencies and bolster respite care and promote caregiver well‐being—both essential to effectively support families of CMC.

## 1 Introduction

Children with medical complexity (CMC) represent a distinct population in paediatric care and are characterized by chronic multisystem conditions, comorbidities, technology dependence, high healthcare utilization and substantial caregiving needs (Gallo et al. 2021; Foster et al. 2025). CMC have experienced improved survival owing to advances in paediatric medicine and technology; however, these developments have shifted the responsibility for intensive and ongoing care to families, particularly to parents who serve as the primary caregivers. In a large‐scale study conducted in the United States, parents of CMC were significantly more likely to report a lack of knowledge about where to seek community support compared with parents of noncomplex children with special health care needs (Bayer et al. 2021). Another quantitative study also reported that parental caregivers of CMC experience particularly high levels of caregiving burden (Akyurek et al. 2026). Furthermore, it is suggested that this burden is not solely attributable to disease management but is also driven by systemic weaknesses within healthcare systems (Geyer and Vessey 2024; Nageswaran and Golden 2018; Glick et al. 2024; Bayer et al. 2025).

Although birth rates have been declining in Japan, the number of CMC has been increasing (Shinjo et al. 2024), partly because Japan has one of the lowest neonatal and under‐five mortality rates worldwide (Collaborators, G. U.‐M. 2021), leading to increased survival of children with severe conditions and, consequently, a growing population of CMC. The Ministry of Health, Labour and Welfare (2016) reported that there were 9403 CMC in 2005; in 2015, the number exceeded 17 078. Further, there were 264 ventilator‐dependent children in 2005 and over 3064 in 2015. Furthermore, existing public services in Japan have been fragmented between welfare and healthcare systems and have not sufficiently met the needs of children with high medical dependency and their parents. As a result, primary parental caregivers of CMC were required to negotiate with multiple separate service providers or administrative offices to access necessary services. This often increased the burden on caregivers, particularly mothers, as child‐rearing responsibilities are predominantly borne by mothers in the Japanese cultural context (Matsuzawa et al. 2020). In response, the Act on Support for Children Requiring Constant Medical Care and Their Families (Act No. 81 of 2021) mandates the establishment of prefectural support centres and the coordination of government agencies across the sectors of health, welfare and education (Ministry of Health, Labour and Welfare 2021). This law aims to provide family‐centred support models for ensuring seamless developmental support as children's needs evolve and for alleviating caregiver burden via the promotion of conditions that facilitate both employment and child rearing. Under this law, in addition to supporting CMC equivalent to those introduced by Olson (2017) (e.g., early developmental services, public school options, financial assistance and respite care), Japan strengthened publicly funded home‐visit services such as physician, nursing, personal care (helper) and bathing assistance services. Moreover, although compulsory education in Japan begins at 6 years of age, after‐school day services have also become widespread. In this system, children attend school in the morning; after school, a transport vehicle accompanied by a nurse picks them up and takes them to the day service facility, where they can stay until around 5:00 PM. In other words, day service facilities are designed to support parents' employment. As these programmes are implemented, local systems are gradually being structured to improve access to home‐ and community‐based resources.

The lived experiences of families with CMC have been illuminated by qualitative research. Parents describe navigating fragmented health systems (Luke et al. 2020), advocating for their children's needs and coping with significant emotional and logistical burden (Allshouse et al. 2018). Parental caregivers report emotional and physical strain from caregiving responsibilities, difficulties securing respite, social isolation and financial burdens. Furthermore, they often experience caregiving as a dynamic tension between resilience and being overwhelmed, with families oscillating between periods of stability and crisis (Hodgson et al. 2024). Research has also highlighted the adaptive strategies employed by parents, such as reframing the definition of caregiving, cultivating informal support networks and engaging in shared decision‐making with health professionals (Barnes et al. 2023; Jacobs et al. 2023). Studies have described how parents cope by nurturing hope, finding small pleasures in daily life and drawing on spiritual and community support alongside the ongoing emotional work required to maintain well‐being under persistent stress (Layshock et al. 2024; Yu et al. 2025). In Japan, parental caregivers report context‐specific challenges and strategies, such as when taking children on excursions, thus reflecting the social and infrastructural barriers unique to the local environment (Kosaka et al. 2023). Although many of these qualitative studies describe coping strategies and challenges of parental primary caregivers, they primarily focus on parents' internal changes, with an emphasis on resilience, emotional efforts and relationships with family members and others.

Collectively, these domestic and international findings suggest that parents are required to manage practical caregiving tasks for their children, coordinate fragmented public services and adjust family roles, representing common challenges across countries. In contrast, Japan is facing a rapid increase in the number of CMC, partly due to having one of the lowest neonatal mortality rates worldwide (Collaborators, G. U.‐M. 2021), and growing attention to CMC has led to the rapid development of support systems. This study aims to clarify what strategies parents with access to public support systems in Japan employ in their daily lives while raising CMC, as well as what challenges remain despite these supports. The novelty of this qualitative study lies in its focus on parental practical strategies and challenges, rather than parents' internal perspectives, within the context of Japan's current public support environment, as explored through qualitative inquiry.

## 2 Methods

This qualitative study adhered to the Consolidated Criteria for Reporting Qualitative Research (Tong et al. 2007) and used an inductive thematic approach (Guest et al. 2012).

### 2.1 Definition of CMC

In this study, CMC were defined as those who require at least one ongoing medical care activity in their daily lives, such as mechanical ventilation, tracheostomy care, oxygen therapy, suctioning, enteral nutrition, dialysis, subcutaneous injections, urinary catheterization, gastrointestinal stoma care, blood glucose monitoring, nebulizer therapy or enema administration.

### 2.2 Participants

Primary parental caregivers who proactively contacted the researcher (M.Y.) after receiving an invitation flyer distributed through 64 visiting nurse agencies in Kyoto and Shiga Prefectures (Kansai, Japan) were enrolled. The inclusion criterion was being a principal at‐home caregiver of CMC < 18 years old for at least 1 year, with the CMC having ongoing dependence on at least one medical technology or care activity, including mechanical ventilation, tracheostomy, oxygen therapy, suctioning, enteral nutrition, dialysis, subcutaneous injections, urinary catheterization, gastrointestinal stoma care, blood glucose monitoring, nebulizer therapy or enema administration. The exclusion criteria were as follows: (1) parents whose child resided in an institution or hospital and (2) families who had transitioned to homecare within the past year (< 12 months).

The recruitment period was from December 2024 to June 2025. The participants voluntarily contacted the researcher (M.Y.), received a detailed explanation of the study and provided written informed consent. An honorarium of USD 20 was offered to compensate the participants for their time. Participant enrollment was concluded upon reaching thematic saturation in the data analysis.

### 2.3 Interview Guide

An interview guide comprising open‐ended questions was employed to elicit the participants' caregiving and parenting experiences. To deepen our understanding of participants' experiences, follow‐up probes were used as appropriate (e.g., What led you to that? and How did you respond at the time?). The interview guide contained the following questions: What support did you receive at the time of discharge?, What support did you use afterward?, What type of support are you currently using?, What types of strategies or adaptations have you used?, What problems or challenges did you face?, and Was there any information you wished to have?

### 2.4 Participant Characteristics

The following descriptive information was collected: the child's age, sex, diagnosis, education, medically necessary devices required for daily use, and medical or welfare services utilized, as well as the parents' age, marital status, roles within the family, employment status and family income.

### 2.5 Data Analysis

Inductive thematic analysis is a method for exploring patterns and meanings in data by systematically organizing content from specific to general and by structuring data into themes and subthemes (Guest et al. 2012). ‘Strategy’ and ‘challenge’ have been conceptualized as paired analytical perspectives in several qualitative studies (Conduru Fernandes Moreira et al. 2022; Rao et al. 2025). In the present study, these perspectives were adopted to clarify how parents navigate and utilize the current care environment, as well as to identify the challenges that persist despite these efforts. By employing this analytical framework, we aimed to facilitate the identification of areas where support from healthcare professionals is needed. The two coders jointly analysed the first case to ensure consistency and shared understanding of coding while discussing their decisions to align and share analytical perspectives on ‘strategies’ and ‘challenges’. The interview data were familiarized through repeated reading of transcripts, followed by open coding to identify meaningful units of text. These codes were then iteratively compared and grouped into broader categories, allowing patterns and themes to emerge inductively. The analysis was conducted consistently using a codebook. Thereafter, for each additional case, they first analysed the data independently and then compared and discussed their results to finalize the analysis. This procedure was repeated each time a new interview was added. In accordance with the principles of thematic analysis, participant sampling was continued until thematic saturation was reached. Specifically, the analysis was concluded when the final few transcripts yielded no new information or insights and when no additional themes emerged.

All interviews were conducted by M.Y. With the consent of the participants, the audio‐recorded interviews were transcribed verbatim. Two coders with extensive qualitative research experience in CMC (M.Y. and Y.S.) verified the accuracy, deidentified the transcripts and analysed the data. Verbatim transcripts were produced in Japanese, and quotations were extracted based on the meaning of their contents. Japanese quotations were translated into English by an interpreter, who took great care to preserve the context and intent of the message. A second translator conducted a back translation (from English to Japanese), and the interviewer (M.Y.) confirmed that the resulting passages (written in Japanese) matched the meanings conveyed by the participants.

## 3 Results

Fifteen parents of paediatric patients were invited to participate in the study via flyers. Thirteen primary caregivers voluntarily contacted the researcher (M.Y.). Among the participants, one withdrew from the study when their child's hospitalization was scheduled during the interview appointment setting, and another was lost to follow‐up during scheduling. The researcher arranged interview appointments in the order that the inquiries were received, and interviews were conducted in the participants' homes, with each interview lasting approximately 1–2 h. By the point of thematic saturation, 13 parent caregivers (11 mothers and 2 fathers) were enrolled. The two fathers attended alongside their wives, but these fathers were not the primary caregivers. A total of 11 interviews were completed with the primary caregivers. The median and interquartile range (IQR) ages of the parents and children were 47.0 [35.0–50.0] and 10.0 [7.0–13.0] years, respectively. The median duration of the interviews was 68.0 [55.0–86.0] minutes. Table 1 presents the demographic characteristics of the participating primary caregivers.

**TABLE 1:** Participant characteristics.

No.	Caregiver					Child										
	Role within the family	Interview time (min)	Age	Employment status	Household members	Sex	Age	Diagnosis	Medically necessary devices	Education	Use of home‐visit physician services	Use of home‐visit nursing services	Use of homecare helper (personal assistance)	Use of home‐visit bathing services	Use of early developmental services	Use of after‐school day services
1	Mother	81	30	Part‐time (10 h/week)	Husband, one CMC, two healthy children	Female	2	GM2 gangliosidosis	Tracheostomy, oral/nasal and tracheostomy suctioning	Daycare	✓	✓			✓	
	Father		30	Full‐time												
2	Mother	58	38	Unemployed	Husband, one CMC	Male	8	Anencephaly	Mechanical ventilator, tracheostomy, supplemental oxygen, oral/nasal and tracheostomy suctioning, nasogastric (NG) tube	Special education school	✓	✓			✓	
3	Mother	86	53	Full‐time	Husband, one CMC, three healthy children	Male	13	Drug‐resistant epilepsy	Oral/nasal suctioning, nasogastric (NG) tube, enema	Special education school		✓	✓	✓		✓
	Father		54	Full‐time												
4	Mother	104	50	Part‐time (12 h/week)	Husband, one CMC, one healthy child	Female	10	Complex congenital heart disease	Oral/nasal suctioning, nasogastric (NG) tube	Special education school	✓	✓				✓
5	Mother	80	47	Part‐time (25 h/week)	One CMC	Male	4	Cerebral palsy	Tracheostomy, oral/nasal and tracheostomy suctioning, nebulizer, nasogastric (NG) tube	None	✓	✓	✓	✓	✓	
6	Mother	68	36	Part‐time (15 h/week)	Husband, one CMC, one healthy child	Female	11	Cerebral palsy	Mechanical ventilator, tracheostomy, oral/nasal and tracheostomy suctioning, nasogastric (NG) tube	Special education school	✓	✓				✓
7	Mother	95	48	Part‐time (9 h/week)	Husband, one CMC, one healthy child	Female	16	Cerebral palsy, epilepsy	Tracheostomy, oral/nasal and tracheostomy suctioning, nebulizer, nasogastric (NG) tube	Special education school	✓					✓
8	Mother	55	50	Unemployed	One CMC, one healthy child	Male	14	Adrenoleukodystrophy	Tracheostomy, oral/nasal and tracheostomy suctioning, nebulizer, nasogastric (NG) tube	Special education school	✓	✓	✓	✓		✓
9	Mother	52	50	Unemployed	Husband, one CMC, one healthy child	Female	10	Crouzon syndrome	Mechanical ventilator, tracheostomy, oral/nasal and tracheostomy suctioning, nasogastric (NG) tube	Special education school	✓	✓	✓	✓		✓
10	Mother	50	40	Part‐time (20 h/week)	Husband, one CMC, one healthy child, maternal grandfather, maternal grandmother	Female	9	Edwards syndrome	Non‐invasive mechanical ventilator, oral/nasal suctioning, nasogastric (NG) tube	Special education school	✓	✓				✓
11	Mother	58	34	Part‐time (20 h/week)	Husband, one CMC, one healthy child, maternal grandfather, maternal grandmother	Male	7	Hydrocephalus	Mechanical ventilator, tracheostomy, oral/nasal and tracheostomy suctioning, nasogastric (NG) tube, enema	Special education school	✓	✓	✓	✓		✓

Abbreviation: CMC, children with medical complexity.

The analysis of the participant interviews yielded the following themes: Theme 1: Skill‐Building, Standardization, and Transition Support; Theme 2: Optimizing the Use of Services and Systems; Theme 3: Homecare Management and Care Coordination; Theme 4: Family Systems and Role Negotiation; Theme 5: Technology Use; Theme 6: Risk Management; Theme 7: Caregiver Rest and Work–Life Balance; and Theme 8: Peer Support and Information Sharing. As the two researchers (M.Y. and Y.S.) progressed with the analysis, it became apparent that the subthemes denoting strategies and challenges were intermingled within themes. Table 2 shows the subthemes for the abovementioned themes. Accordingly, the subthemes were classified as a strategy (ST) or challenge (CH). Table 3 lists examples of quotations by subtheme.

**TABLE 2:** Themes and subthemes.

Themes	Subthemes
Skill‐building, standardization and transition support	[ST] Utilization of trial overnight‐stay programmes in hospital
	[ST] Drawing on prior cases
	[ST] Building the ability to assess the child's condition
	[CH] Insufficient discharge teaching
	[CH] Shortage of paediatric home‐visit nurses
Optimizing the use of services and systems	[ST] Maximize service use
	[ST] Plan services ahead of development
	[ST] Create child‐friendly environments
	[ST] Using one programme as a gateway to connect with other programmes
	[CH] Difficulties arising from support needs that change with age
	[CH] Services are not comprehensive
	[CH] Insufficient information for transition
Homecare management and care coordination	[ST] Importance of consultation workers
	[CH] Lack of interagency coordination
Family systems and role negotiation	[ST] Distributing care within family
	[CH] Limited parental bandwidth for siblings' upbringing
Digital technology use	[ST] Utilizing telemedicine
Risk management	[ST] Building connections with local community members
	[ST] Planning for disasters
Caregiver rest and work–life balance	[ST] Importance of short‐hour employment
	[CH] Lack of respite for primary caregiver
	[CH] No backup caregiving
Peer support and information sharing	[ST] Sharing information among peers

Abbreviations: CH: challenge; ST: strategy.

**TABLE 3:** Representative quotations by subtheme.

Subtheme	Quotation
Theme 1/ [ST] Utilization of trial overnight‐stay programmes in hospital	We took turns staying with our child in the hospital on a one‐day rotation. My husband was admitted with our son as well so he could learn the care. During the day, Grandpa and Grandma took over. Each of us tried doing the suctioning and other tasks on our own. For about a month, it felt like a training camp—the four of us rotated in and out, practicing until we felt confident.
Theme 1/ [ST] Drawing on prior cases	I really wanted to bring my child home, but when I pictured bringing home a kid on a ventilator, I got super anxious. So I talked to a nurse, and she arranged for me to meet, in a hospital room, some moms who had kids like mine. One of them even said, ‘You can come see our home if you want’, so before discharge we went and took a look at her home. Just that alone really calmed me down—I thought, okay, we can do this. My husband came too, and the two of us could finally picture what it would be like to bring our child home.
Theme 1/ [ST] Building the ability to assessment the child's condition	You start to get a feel for it—like, ‘Yeah, this looks like we need to get them admitted.’ When you are watching your child all the time, it gradually becomes clearer. It's really hard at first, right? But keeping a close eye on them every day is so important.
Theme 1/ [CH] Insufficient discharge teaching	At some hospitals, there's basically no family training when you are discharged. The differences are huge. I want the nurses to actively encourage not just me, but also my husband and the grandparents, to learn to do suctioning.
Theme 1/ [CH] Shortage of paediatric home‐visit nurses	From what I've heard, there are hardly any home‐visit nursing agencies that can actually take pediatric cases.
Theme 2/ [ST] Maximize service use	We use every in‐home service we can: a visiting nurse, a speech therapist, a physical therapist, and a home‐visit doctor. We also get home‐visit bathing—two helpers come on Monday, Tuesday, Wednesday, Thursday, and Friday.
Theme 2/ [ST] Plan services ahead of development	When my child was little, I felt we should use every support program we could. Once they start grade school and get heavier, trying to set things up then gets really hard and takes a lot of time. So from the very beginning—right after discharge—I wanted to line up a good amount of welfare services, lean on the nurses where we can, and still keep a little time for myself.
Theme 2/ [ST] Create child‐friendly environments	The first thing I noticed was the sound of kids crying and laughing. In special‐needs facilities, there aren't many kids who vocalize a lot, so you mostly hear adults' voices. At first, that was a huge overload for him. When he started going to a regular daycare, he'd come home wiped out and just sleep. He even slept most of the time at daycare, and I worried—is he okay? But little by little, he got used to it. Being part of a group of kids his own age has been an amazing experience.
Theme 2/ [ST] Using one programme as a gateway to connect with other programmes	Through our home‐visit nursing agency, we started using an after‐school day service run by the same company, so we were able to have the same familiar nurses across both services.
Theme 2/ [CH] Difficulties arising from support needs that change with age	After graduation, they are treated as an adult with a disability instead of a child, so the laws and the services they can use switch over. They used to go to a special support school, but once they graduate, there's no ‘school’ to go to—and everything changes really fast.
Theme 2/ [CH] Services are not comprehensive	For older adults, there are lots of service options tailored to the person. Even within home‐visit nursing, you can choose places that are strong with rare diseases or that have specialist nurses. For children, the options are extremely limited. It really comes down to coordination—and that part just is not being done.
Theme 2/ [CH] Insufficient information for transition	After they turn 18, there's just not enough information. It feels like the only way is to collect and piece together stories from more experienced moms—like, ‘That life‐care/day program over there is good’, and so on.
Theme 3/ [ST] Importance of consultation workers	I run a lot of things by our coordinator, and they have been a huge help.
Theme 3/ [CH] Lack of interagency coordination	It takes forever to get the info—we have to work so hard just to find what we need. I'll ask other moms, then try the city office … nope, not there, not here … oh, maybe here. In the end, we only get there with help from others.
Theme 4/ [ST] Distributing care within family	Grandpa and Grandma can do suctioning too, and since they are involved every day, the fear has really faded. Of course, they think the kid is adorable, and over time we have all gotten used to it. It made me realize how important it is for everyone to be involved.
Theme 4/ [CH] Limited parental bandwidth for siblings' upbringing	My husband handles the drop‐offs for our kid's lessons. But if the other one has a different class too, we just cannot manage it.
Theme 5/ [ST] Utilizing telemedicine	My daughter's nail has not been growing back for over a month. I emailed the doctor a photo, and they replied, ‘Could you send one from a different angle?’ After that, they said it was paronychia. I told them I was putting on an ointment we already had at home and asked if that was okay, and they said, ‘Yes, keep using it.’ It was all done by telemedicine. If she gets a bit of a fever or needs medication, they can prescribe it online right away and even have the meds delivered. It's so convenient.
Theme 6/ [ST] Building connections with local community members	My landlord lives nearby, and they have been really thoughtful. They told me, ‘If getting your CMC child in and out of the car is hard, feel free to use the parking lot’, and, ‘If you ever need power outdoors, you can use this outlet here.’ I'm not sure how much that will truly help in every situation, but it made me realize it's important to speak up and let people know we have a child with medical complexity living here.
Theme 6/ [ST] Planning for disasters	When we talk as a family about what to do in a disaster, it comes up a lot. Honestly, we probably cannot just grab our child and run, so our plan is more like: if the power goes out, we do X, Y, Z. And if my family cannot get in touch with me right away, I've told them to at least let someone know our child is here—call an ambulance or get help. If we are able to move, we'll head to the nearby hospital and try to use their power. But in reality, plans like this are pretty vague, right? That's why I feel it would really help if the community knew that a child on a ventilator lives here—then they could step in faster when something happens.
Theme 7/ [ST] Importance of short‐hour employment	Since the day service started taking my child on Saturdays, I've been able to pick up a part‐time job. That was a really big step forward for me. It exceeds the value of the income.
Theme 7/ [CH] Lack of respite for primary caregiver	We were talking about how the hardest thing is when a parent has a high fever—like 104°F—but still has to care for a CMC child. I once caught a stomach bug at the same time as my son. He kept vomiting over and over, I was throwing up too, and there I was trying to cook something and run the laundry because all the clothes were soiled. I get that in sudden illnesses it's hard to find help. But I still want a chance to breathe. I'd like to use respite care—just a little break. Even two or three days would be enough to step away from my child and take care of my own health.
Theme 7/ [CH] No backup caregiving	Home‐visit nursing is often just short slots—like only for bath time. And they usually come while the mom is still there, so it does not really give her any respite. Ideally, they should be able to step in for the mom, you know? If they could swap in when the mom needs to go shopping or pick up a child, that would be a huge help for mothers.
Theme 8/ [ST] Sharing information among peers	I have a CMC friend of my son's who had a laryngotracheal separation, and I saw how much their hospitalizations went down. Knowing that made it easier for us to make the decision.

Abbreviations: CH: challenge; ST: strategy.

### 3.1 Theme 1: Skill‐Building, Standardization, and Transition Support

During the transition from inpatient treatment to homecare, parents of CMC systematically acquired the practical skills required for daily medical care, observation and management. The key strategies included rooming‐in practice (24‐h homecare rehearsal within the hospital), learning from established homecare cases involving similar CMC and the deliberate development of caregiving competence (ST). However, not all parents were able to acquire these skills to a sufficient degree. Several participants perceived that discharge education was inadequate, and the scarcity of paediatric‐trained home‐visit nurses contributed to anxiety about post‐discharge care (CH).

### 3.2 Theme 2: Optimizing the Use of Services and Systems

Parents of CMC assumed dual responsibility for ongoing medical management and fostering their child's development. To optimize everyday life at home, they proactively leveraged available public services, anticipated developmental needs and initiated services preemptively (ST). They also created developmentally appropriate child‐friendly environments to promote growth and described how engagement with one service often facilitated access to additional support (ST). Conversely, parents reported the following persistent challenges: service needs changed with the child's age, available support was fragmented rather than comprehensive, and services and information on transition‐to‐adult care were insufficient (CH).

### 3.3 Theme 3: Homecare Management and Care Coordination

Children received nursing and personal care across multiple settings, including the home, special needs schools, preschools/elementary schools and day services, and families actively utilized home‐visit medical care, nursing, rehabilitation and personal care services (ST). The key factor enabling this multisite care was the involvement of a consultation worker/care coordinator who helped organize services and facilitated interagency collaboration (ST). Parents identified gaps in information sharing among agencies and settings as a key challenge that hindered seamless coordination and continuity of care (CH).

### 3.4 Theme 4: Family Systems and Role Negotiation

Families in the child‐rearing phase must manage multiple demands, including employment, household responsibilities and parenting. For households raising CMC, sustained collaboration is essential. Families intentionally distribute caregiving tasks within the household to prevent excessive burden on the primary caregiver (ST). Moreover, parents reported limited bandwidth for the care and upbringing of siblings, thus underscoring the difficulty of balancing attention and resources across multiple children (CH).

### 3.5 Theme 5: Digital Technology Use

In Japan, technology has become increasingly embedded in home‐based care for CMC. Within this theme, only strategies were identified. Families reported leveraging telemedicine and other online modalities, which offer practical benefits such as reduced travel burden and timely access to clinical consultation and follow‐up (ST).

### 3.6 Theme 6: Risk Management

The Japanese public has a high level of awareness of natural disaster risk (e.g., earthquakes). For children who are reliant on medical devices, such as mechanical ventilators, rapid evacuation from home is inherently difficult. Parents exclusively described strategy‐oriented responses, such as implementing self‐directed preparedness measures and proactively notifying local residents and first responders (e.g., fire and police departments) about the presence of CMC to facilitate timely assistance during emergencies (ST). No distinct challenges were identified, and the actions described were preventive in nature.

### 3.7 Theme 7: Caregiver Rest and Work–Life Balance

Within this theme, challenges predominated over strategies. Parents noted that brief periods of employment while the child attended day services functioned as vital social engagement and psychological respite, benefits that exceeded the value of income (ST). At the same time, they expressed a clear unmet need for restorative rest for primary caregivers and reported that current home‐visit nursing and personal care services did not adequately provide respite or rejuvenation (CH).

### 3.8 Theme 8: Peer Support and Information Sharing

Peers play a pivotal role in the exchange of information. Parents actively shared diverse experience‐based information regarding treatment, daily care and available support, thus generating mutual benefits and creating a sense of solidarity within the family association (ST).

## 4 Discussion

### 4.1 Global Challenges Identified Across the Eight Themes

The family‐inclusive CMC law enacted in Japan in 2021 clarified municipal duties and increased funding, thus providing a uniquely supportive policy environment. However, the identified strategies and difficulties mirror those documented internationally, indicating that core challenges, such as coordination gaps, limited respite and inadequate transition‐to‐adult care support, persist despite the enactment of advanced policies. This alignment underscores the fact that many issues are global in nature and highlights the clinical importance of solutions that extend beyond entitlement expansion to address implementation and coordination issues at the point of care.

This study revealed that overnight clinical research stays during hospitalization and opportunities for interaction with families in similar situations functioned as strengths when available but became sources of difficulties when insufficient. This finding is consistent with that of Glick et al. (2024) regarding the challenges in understanding and implementing discharge instructions. At the same time, the shortage of paediatric‐trained home‐visit nurses remains a significant barrier after discharge (Kemp et al. 2021; Nageswaran and Golden 2018), and the findings of the current study underscore this persistent gap. Narratives describing how participation in regular daycare and community activities promoted children's development align with the findings of Foster et al. (2017), who highlighted the importance of a ‘child‐centred setting’. However, our results also indicated discontinuities and insufficient information regarding age‐ and transition‐related support, echoing the study of Joly (2016) on the vulnerability of transition periods.

Our study shows that consultation (care coordination) workers play a central role in organizing services for CMC, thus underscoring the importance of specialized coordination roles. At the same time, the lack of interagency information sharing is consistent with the findings of Jacobs et al. (2023) regarding inadequate collaboration in community care. Therefore, there is a need for stronger coordination among the service providers involved in home‐based care. Narratives about distributing caregiving responsibilities within families are consistent with the findings of Teicher et al. (2023) on intrafamilial role sharing and mutual support, highlighting this as a key coping strategy.

The utilization of telemedicine and online prescription services in our study corresponded with that in Frush et al. (2023), who emphasized information support as a means of reducing emergency department use and enhancing convenience in homecare.

In addition, this study identified preparedness for disasters as part of caregivers' everyday strategies. Participants reported that they had engaged in concrete discussions with neighbours and public agencies regarding how to respond in the event of an earthquake. This may be related to the fact that Japan is a country frequently affected by natural disasters such as earthquakes. A review by Smith et al. (2024) highlighted the importance of utilizing community networks and peer support prior to disasters. It is noteworthy that similar practices were clearly identified as caregiving strategies in the present Japanese context.

Effendy et al. (2022) reported on the desire of parents to continue providing care while maintaining the possibility of working from home. Additionally, in this study, some parents expressed a desire for reducing employment hours (short working hours) by leveraging available support schemes. An analysis of parental narratives indicated that parents sought to maintain their own well‐being through work. Therefore, it is important to recognize that employment functions not only as a source of income but also as a means of social participation and self‐realization for parents, thus reaffirming these factors as internationally recognized challenges. Caregivers frequently described being unable to secure time for themselves, consistent with the findings of Layshock et al. (2024) on stress coping and self‐preservation. In particular, caregivers' urgent plea for ‘just a few hours of rest’ underscores the need to expand social support services, such as home‐based assistance and formal respite care.

Finally, peer networks among families of CMC provided support by reducing social isolation and enhancing engagement and confidence; this finding aligns with that of Allshouse et al. (2018) on the benefits of family‐to‐family and parent‐to‐parent support. These networks also aided in the navigation of issues involved in care planning and collaboration. In the current study, peer support emerged as a vital factor in preventing caregiver isolation and fostering positive adaptation.

The study findings reveal the following key clinical priorities for supporting parents who care for CMC at home: expanding the paediatric‐trained home‐visit nursing workforce, strengthening support for transition‐to‐adult care support, enhancing coordination among home‐ and community‐based agencies, bolstering respite care and promoting caregiver well‐being. Addressing these priorities is essential for effectively supporting families of CMC.

### 4.2 Contextual Insights From the Japanese Setting

Overall, the findings yielded novel insights specific to the Japanese context. First, approximately half of the children in this study were dependent on mechanical ventilation, and more than half had a tracheostomy. These findings indicate that many of the children had high levels of medical dependency, requiring continuous monitoring and hands‐on care by caregivers. Regarding the participants, parents were recruited as primary caregivers. As a result, the study sample consisted of 11 mothers as primary caregivers. The two fathers participated together with the mothers in the interview; however, they were not the primary caregivers. Among the 11 mothers, only one was employed full‐time, whereas the others were either engaged in very short working hours or were unemployed. The Act on Support for Children with Medical Complexity, enacted in Japan in 2021, includes the indirect support of parental employment through the utilization of social systems as one of its objectives (Ministry of Health, Labour and Welfare 2021). However, considering the characteristics of both the participants and their children in this study, mothers of children with high medical dependency were largely unable to engage in employment. Japan has a relatively high rate of part‐time employment among women (39%) compared with part‐time employment rates in countries such as Switzerland (15%), the United States (16%) and South Korea (23%), whereas the proportion of women in full‐time employment remains comparatively low (The Japan Institute for Labour Policy and Training 2024). Within the Japanese cultural context, it is relatively common for mothers to engage only in limited working hours, and this issue has been widely discussed from a socioeconomic perspective. At the individual level, findings related to Theme 7: Caregiver Rest and Work–Life Balance indicated that caregivers desired short‐duration employment as a means of maintaining their well‐being, suggesting that more careful and nuanced discussions are needed regarding employment for parents of children with high medical dependency.

The inclusion of disaster preparedness within Theme 7 ‘Risk Management’ in this study is considered to reflect the Japanese context, where disasters are frequent and preparedness is a routine necessity. Because mechanical ventilation requires a stable power supply, high‐quality power generation systems are essential. According to a study conducted in Japan among parents of children using home mechanical ventilation, more than 60% had secured a power supply to ensure the continued use of medical devices during power outages (Matsumoto et al. 2022). Advanced medical devices such as ventilators require a stable power supply. In Japan, municipalities have implemented measures such as distributing high‐quality backup generators and developing individualized disaster response plans to ensure a reliable power supply during emergencies. Although this study identified individual‐level strategies in Japan, disaster preparedness measures should vary depending on regional climate and geographical characteristics. From a public health perspective, it is essential to develop context‐specific strategies tailored to local conditions.

This study has a few limitations. First is the bias stemming from the composition of the participants. Our findings reflect the situation in a single region of Japan, a regional city and its surrounding municipalities and are not representative of the situation at the national level. The second limitation is that all primary caregivers were mothers, even though some fathers participated. This finding reflects the situation in Japan, where mothers are typically the primary caregivers; however, it cannot be assumed that fathers never serve as the primary caregivers of CMC. Therefore, the limited representation of participants in this study may have influenced the results. For example, the pursuit of work–life balance identified in Theme 7 might have yielded different themes from a father's perspective. Further research focusing on fathers is warranted. The third limitation is the limited generalizability inherent in qualitative research methods. Therefore, prospective cohort studies and subsequent investigations that build upon the findings of the current study are warranted.

## Author Contributions

**Miku Yamaguchi:** conceptualization, methodology, investigation, formal analysis, writing – original draft, writing – review and editing, visualization, data curation, supervision. **Yuki Suzuki:** methodology, formal analysis, data curation, supervision, writing – review and editing.

## Funding

This study was supported by a Grant‐in‐Aid for Scientific Research (Grant No. 22K17506) from the Japan Society for the Promotion of Science of the Ministry of Education, Culture, Sports, Science and Technology.

## Ethics Statement

This study was approved by the Institutional Medical Ethics Committee of the Kyoto Prefectural University of Medicine (Approval No. ERB‐E‐530‐1).

## Consent

Written informed consent was obtained from all participants prior to the interviews and audio recordings.

## Conflicts of Interest

The authors declare no conflicts of interest.

## Data Availability

De‐identified interview transcripts are not publicly available due to privacy and confidentiality restrictions. The coding framework, codebook and analytical memos are available from the corresponding author upon request.
